# Spousal age difference and associated predictors of intimate partner violence in Nigeria

**DOI:** 10.1186/s12889-018-5118-1

**Published:** 2018-02-02

**Authors:** Ayo Stephen Adebowale

**Affiliations:** 1 Department of Epidemiology and Medical Statistics, Faculty of Public Health, College of Medicine, University of Ibadan, Ibadan, Nigeria; 2 Centre for Actuarial Research (CARe), University of Cape Town, Cape Town, South Africa

**Keywords:** Spousal age difference, Intimate partner violence, Couples, Nigeria

## Abstract

**Background:**

The growth in Intimate Partner Violence (IPV) cases among couples in Nigeria has been significant in recent years. Victims, which are often females, face numerous health challenges, including early death. I examined the linkages between spousal age differences and IPV in Nigeria.

**Method:**

The couples recode data section of the 2013 Nigeria Demographic Health and Survey was used (*n* = 6765). Intimate partner violence was measured using 13-item questions. Data were analyzed using the logistic regression model (α = .05).

**Results:**

The mean spousal age difference was 8.20 ± 5.0 years. About 23.5, 18.0, 13.5 and 4.7% of couples surveyed had experienced some form of IPV, emotional, physical and sexual violence respectively. Also, IPV prevalence was 27.0, 23.7, 22.0 and 18.7% among couples with age differences of 0–4, 5–9, 10–14 and ≥15 years respectively; this pattern was exhibited across all domains of IPV. Among women who experienced physical violence, 20.5% had only bruises, 8.0% had at least one case of eye injuries, sprains and/or dislocations, and 3.7% had either one or more cases of wounds, broken bones or broken teeth. The identified predictors of IPV were: family size, ethnicity, household wealth, education, number of marital unions and husband drinks alcohol. The unadjusted likelihood of IPV was 1.60 (C.*I* = 1.30–1.98, *p* < 0.001) and 1.35 (C.*I* = 1.10–1.64, *p* < 0.01) higher in households where the spousal age difference was 0–4 and 5–9 years respectively, than the likelihoods among those with a spousal age difference ≥ 15 years, but the strength of the association weakens when other variables were included in the model.

**Conclusion:**

The level of IPV was generally high in Nigeria, but it reduced with increasing spousal age difference. This study underscores the need for men to reach a certain level of maturity before marriage, as this is likely to reduce the level of IPV in Nigeria.

## Background

Violence against women is “any act of gender-based violence that results in, or is likely to result in, physical, sexual or psychological harm or suffering to women, including threats of such acts, coercion or arbitrary deprivation of liberty, whether occurring in public or in private life” [1]. Intimate partner violence (IPV) in the context of this study is any act of violence against women perpetrated by their husbands [2]. Intimate partner violence mirrors entrenched gender inequality, constitutes an extreme form of discrimination against women and has long-term consequences [2]. Despite the level of modernization achieved at the global level, many married women are still being maltreated by their husbands. The adoption of zero violence against women by their husbands is essential to building stronger families, creating robust economies, and accomplishing internationally agreed upon Sustainable Development Goals (SDGs) such as Agenda 5, namely, to achieve gender equality and empower all women girls [3].

Literature on IPV in low-income countries has focused largely on Asia [4–6] and some parts of sub-Saharan Africa [7, 8], but has thus far ignored Nigeria [9–11]. In Africa, reported IPV is more common in Southern Africa and Central Africa and increasingly in Nigeria [12]. The upsurge in IPV in Nigeria can be attributed to several factors, including harsh economic conditions, social networks, immaturity on the part of the couple and childlessness in marriage [12, 13]. Victims of IPV face bodily defects, infections, unintended pregnancies, unsafe abortions, pregnancy complications and untimely death [2, 9, 14, 15]. In spite of the benefits of marriage, such as providing optimal conditions for childbearing and rearing, promoting a healthier life, the provision of emotional support, and the reduction of depression, the problems associated with marriage could be devastating if domestic violence is prevalent [9, 14, 15].

Spousal healthy relations and a successful married life are the result of many factors. One salient factor, which usually goes unnoticed is spousal age difference. Spousal age difference refers to the difference between the ages of wife and husband. The tradition of male dominance in marriage is still prevalent in Nigeria, where, apart from a few exceptions, men marry women that are younger than they are [16]. This could be one of the ways of creating an avenue for men in exercising power as the family head. The long-standing poor economic state of Nigeria has heightened the postponement of marriage among men [16]. This age gap in marriage poses several problems: for instance, there may be differences in maturity and differences in opinions; the partners’ sexual life may be affected at a later stage, if husband is much older than the wife; early planning for children might be necessary if husband is much older; ageing and early widowhood are also problems associated with a wide spousal age gap [17, 18]. It is always possible that a couple may influence each other to adopt new interests, but an age gap in the relationship can compromise this. Moreover, an age disparity in marriage is positively related to decreased longevity, particularly for women [18], because of the increased IPV that might result when women in such a relationship refuse or negotiate sex [19]. In other words, if the younger woman refuses to have sex with the older man, she is more likely to be beaten, which might reduce her lifespan.

Women bear the overwhelming burden of IPV in Nigeria. The 1993 United Nations General Assembly resolution recommends the promotion of research, especially regarding domestic violence, relating to the prevalence of different forms of violence against women, and encourages research on the consequences of violence against women [1]. Thus, it is imperative to document the policy relevance of the relationship between spousal age difference and IPV in an effort to reduce its prevalence and improve maternal health in Nigeria. The objectives of this study are thus to examine the connection between the age gap of partners in a marital union and IPV, and to do so across three domains of IPV, namely, emotional, physical and sexual violence.

### Literature review and theoretical framework

A growing number of research has been conducted into the factors associated with IPV and its three domains [4, 12, 13]. A multi-country study by the World Health Organization confirmed that IPV is widespread across all countries with the prevalence ranging between 4% and 75% [20]. A comparative study from nine countries including, sub-Saharan Africa, Asia and South America also found that the percentage of women who had recently experienced physical violence or sexual violence ranged from 18 to 48% and 4 to 17% respectively [21]. In another study, physical or sexual IPV reported by currently married women ranged from 17% in the Dominican Republic to 75% in Bangladesh [22]. Several risk factors of IPV, such as being young in age as a woman, low level of education, poverty, place of residence, exposure to violence between parents, sexual abuse during childhood and a general acceptance of violence have been consistently identified in the literature [4, 6, 23].

In sub-Saharan Africa, the IPV prevalence ranges from 30.5% in Nigeria to 43.4% in Zimbabwe, 45.3% in Kenya, 45.5% in Mozambique, 53.9% in Zambia and 57.6% in Cameroun [12]. A study conducted in Tanzania reported a prevalence of physical and/or sexual IPV of 61% among women; this prevalence varied by socio-demographic characteristics, showing much higher prevalence rates among younger women, women with young partners and less educated women [7]. In a study conducted in rural Uganda on IPV in 2016 ranged from 6.49% (severe physical abuse) to 31.99% (emotional abuse), and severe physical IPV was significantly associated with divorce/separation [8]. A hospital-based study in Kano, Nigeria showed an IPV prevalence of 42.0%, with 46.6%, 29.0% and 21.9% of participating women having experienced emotional/psychological violence, physical violence and sexual violence respectively [9]. Marriage type and alcohol consumption by the partner were found to be important predictors of IPV in studies conducted in different parts of Nigeria [9, 14]. A household survey of IPV in two Nigerian states found that IPV prevalence during the last pregnancy was 22% and 9% in Cross River and Bauchi states respectively, with the risk being lower among poor women with more educated partners [10]. In a hospital-based study in south-western Nigeria, the prevalence of IPV associated with infertility among women was 31.2%, and factors implicated as IPV predictors were unemployment and prolonged duration of marital infertility [13].

In order to understand IPV within a family violence structure, different frameworks have been used by sociologists. The family violence perspective views conflict between family members as universal and inevitable, and holds the view that most family violence is not the result of individual pathology but that it is a “normal part of family life in most societies” [24]. Straus was the first to propose the application of systems theory to family violence, positing that violence between members of a family is a “systemic product rather than a chance aberration or a product of inadequate socialization or a warped or psychotic personality” [25]. In the context of family violence, Straus theorized that positive feedback from interactions within the system increases or amplifies violence, while negative feedback decreases or controls violence [25]. Giles-Sims advocates that many family structure characteristics, such as socialization, time spent together, and stress level, all have an impact on the potential for violence in the system [26].

A nested ecological theory by Dutton and Nicholls states that more precise variables are viewed as ‘nested in’ broader variables [27]. This theory focuses on the complex and interrelated networks of systems that influence behavior, including violent behavior; it listed the family unit as the immediate context that surrounds the individual as one of the four causative factors of violence. The social exchange theory posits that violence occurs at the family level if the benefits of violent behavior outweigh the risks [28]. Using this theory as a foundation for understanding IPV, Gelles asserted that, to reduce the occurrence of violence within the family, the rewards of using violence must be decreased [28]. The feminist perspective of IPV as propounded by Dobash and Dobash is that wife abuse is an expression of male domination over women, and stressed that the patriarchal domination of women through wife abuse is held over from the long cultural history of legally authorized male subordination, abuse, and outright ownership of women [29].

## Methods

This study was conducted in Nigeria, a country in the West African sub-region. By geographic definition, Nigeria is predominantly a rural country and the level of illiteracy is high. A national survey found that the median age at first marriage for men and women was 18.1 and 27.2 years respectively [16].

The study was cross-sectional population-based and used weighted 2013 Nigeria Demographic and Health Survey data, with a focus on the couple recode section of the data. It was a nationally representative sample, with participants selected across all the states in Nigeria. The survey used as a sampling frame the list of enumeration areas (EAs). Administratively, Nigeria is divided into 36 states and a Federal Capital Territory (FCT). These states are subdivided into 774 LGAs, and each LGA is divided into localities. Each locality was subdivided into EAs. The EAs, which were the primary sampling units (PSU), were used as a cluster with a minimum size of 80 households. The sample was selected using a stratified three-stage cluster design, consisting of 904 clusters, 372 in urban areas and 532 in rural areas. A complete listing of households and a mapping exercise were carried out in each cluster, with the resulting lists of households serving as the sampling frame for the selection of households. All regular households were listed. Global Positioning System (GPS) receivers were used to calculate the coordinates of the sample clusters. A fixed sample of 45 households was selected per cluster. In this study, a sample of one eligible woman in each household was randomly selected to be asked questions regarding domestic violence [16].

Only couples with complete information on variables that were used for the generation of IPV and age difference were included in the study. A small number of cases, where women were older than their husbands, were excluded. Thus, the total sample of couples included in the study was 6765. Intimate partner violence was created using information on the following items:

**Table:** 

		*Response*	
*S/N*	*Items*	*No*	*Yes*
1.	Ever been humiliated by husband/partner	0	1
2.	Ever been threatened with harm by husband/partner	0	1
3.	Ever been insulted or made to feel bad by husband/partner	0	1
4.	Ever been pushed, shaken or had something thrown by husband/partner	0	1
5.	Ever been slapped by husband/partner	0	1
6.	Ever been punched with fist or hit with something harmful by husband/partner	0	1
7.	Ever been kicked or dragged by husband/partner	0	1
8.	Ever been strangled or burnt by husband/partner	0	1
9.	Ever been threatened with knife/gun or other weapon by husband/partner	0	1
10.	Ever had arm twisted or hair pulled by husband/partner	0	1
11.	Ever been physically forced into unwanted sex by husband/partner	0	1
12.	Ever been forced into other unwanted sexual acts by husband/partner (threats)	0	1
13.	Ever been physically forced to perform sexual acts respondent did not want to	0	1

The items 1–3, 4–10 and 11–13 above constitute the emotional violence (EV), physical violence (PV) and sexual violence (SV) domains respectively, as measured by the demographic health and survey [16]. In order for women to live healthy lives in marriage, they should not be on the receiving end of violence. Therefore, any woman who chose yes for at least one of the responses on the item list was considered to have experienced violence. This is also the situation for the three domains of violence: emotional (yes to at least one of the 1st to 3rd items), physical (yes to at least one of the 4th to 10th items on the list) and sexual violence (yes to at least one of the 11th to 13th items) as indicated below. IPV, EV, PV and SV are the dependent variables.

**Table:** 

$$ \boldsymbol{IPV}=\left\{\begin{array}{c} No\kern4.25em if,y=0\\ {} Yes\kern1.5em if,1\le y\le 13\end{array}\right. $$	$$ \boldsymbol{EV}=\left\{\begin{array}{c} No\kern4.25em if,y=0\\ {} Yes\kern1.5em if,1\le y\le 3\end{array}\right. $$	$$ \boldsymbol{PV}=\left\{\begin{array}{c} No\kern4.25em if,y=0\\ {} Yes\kern1.75em if,1\le y\le 7\end{array}\right. $$	$$ \boldsymbol{SV}=\left\{\begin{array}{c} No\kern4.25em if,y=0\\ {} Yes\kern1.75em if,1\le y\le 3\end{array}\right. $$

*Where “y” is the number of items of violence previously experienced*

The main independent variable was spousal age difference, which was generated by subtracting the ages of the women from the ages of their husbands. These age gaps were later categorized as 0–4, 5–9, 10–14 and ≥15 years with a view to examining which spousal age gap inhibits or promotes IPV, other than using age difference as a continuous variable in the analysis. Other independent variables include the following: number of living children, residence, ethnicity, religion, household wealth, education, marriage type, husband drinking alcohol, husband’s education, number of marital unions and empowerment.

Data were analyzed using t-test, Chi-square and logistic regression at 5% level of significance. The t-test was used to determine the mean age difference by IPV. The Chi-square was used to examine the association between IPV, EV, PV, SV and socio-demographic characteristics, including spousal age difference. Due to the dichotomous nature of each of the dependent variable, we used the logistic regression model to identify the predictors of IPV and other domains of violence. The selection of variables into the logistic regression was done by running a bivariate analysis of the dependent variable and an independent variable. Thereafter, any statistically significant variable based on the Wald test from logistic regression and a *p*-value cut-off point of 0.25 was included in the first model. In the iterative process of variable selection, covariates were removed from the model if they were not significant (at 0.1 alpha level) and not a confounder. At the end of the iterative process of deleting, refitting, and verifying, the final model was fitted.

Two models (unadjusted and adjusted) were used to examine the relationship between age difference and IPV, EV, PV, SV. The unadjusted models (Eq. 1) were the bivariate analysis of the relationship between IPV, and each of the independent variables, while the adjusted models (Eq. 2) included other variables as control.

1 $$ \log \left\{\frac{p_i}{1-{p}_i}\right\}={\upbeta}_0+{\upbeta}_{1\mathrm{i}}{\mathrm{x}}_{1\mathrm{i}} $$

2 $$ \log \left\{\frac{p_i}{1-{p}_i}\right\}={\upbeta}_0+{\upbeta}_{\mathrm{pi}}{\sum}_{i=1}^n{\mathrm{x}}_{\mathrm{pi}} $$

β_0_, β_pi_ are the regression parameters, x_i_ are the independent variables, and p_i_ represents the proportion of women who have experienced IPV in the i^th^ category of a particular variable.

## Results

In Table 1, the data show that the mean age of husbands was higher than that of their wives across all the domains of violence; the mean age of husbands of women who experienced IPV was higher than those who did not experience IPV. This pattern was also observed among all three domains of violence. The mean age of husbands and wives was 36.3 ± 7.4 and 28.1 ± 7.1 years respectively, while the mean spousal age difference was 8.20 ± 5.0 years. The mean spousal age difference was significantly lower among couples where women had experienced IPV (7.72 ± 4.) than among those who did not (8.34 ± 5.1). This is also the pattern for emotional, physical and sexual violence.

**Table 1:** Percentage Distribution of Couples by mean age of the Husband age, mean age of the Wife, mean spousal age difference according to domains of violence

Violence domain	Experienced violence status	Husband’s mean age	F-value	Wife’s mean age	F-value	Mean age difference	F-value
Indicators		$\left(\overline{\mathrm{x}}\pm \upsigma \right)$	(*p*-value)	$\left(\overline{\mathrm{x}}\pm \upsigma \right)$	(*p*-value)	$\left(\overline{\mathrm{x}}\pm \upsigma \right)$	(*p*-value)
Total		36.3 ± 7.4		28.1 ± 7.1		8.20 ± 5.0	
IPV	No	36.1 ± 7.4	4.138^c^	27.8 ± 7.2	27.124^a^	8.34 ± 5.1	18.284^a^
	Yes	36.6 ± 7.2	(0.042)	28.8 ± 6.7	(< 0.001)	7.72 ± 4.8	(< 0.001)
Emotional	No	36.2 ± 7.5	0.912	27.9 ± 7.1	12.801^a^	8.30 ± 5.1	5.452^c^
	Yes	36.4 ± 7.0	(0.340)	28.7 ± 6.6	(< 0.001)	7.82 ± 5.0	(0.020)
Physical	No	36.2 ± 7.4	5.888^c^	27.8 ± 7.1	36.715^a^	8.32 ± 5.1	8.349^b^
	Yes	36.8 ± 7.3	(0.150)	29.4 ± 6.8	(< 0.001)	7.56 ± 4.8	(0.002)
Sexual	No	36.3 ± 7.4	0.367	28.1 ± 7.1	0.097	8.20 ± 5.1	0.650
	Yes	36.0 ± 7.4	(0.544)	27.9 ± 7.0	(0.755)	8.07 ± 4.7	(0.206)

*IPV* Intimate partner violence

^a^Significant at 0.1%; ^b^Significant at 1.0%; ^c^Significant at 5.0%

**Fig. 1:**
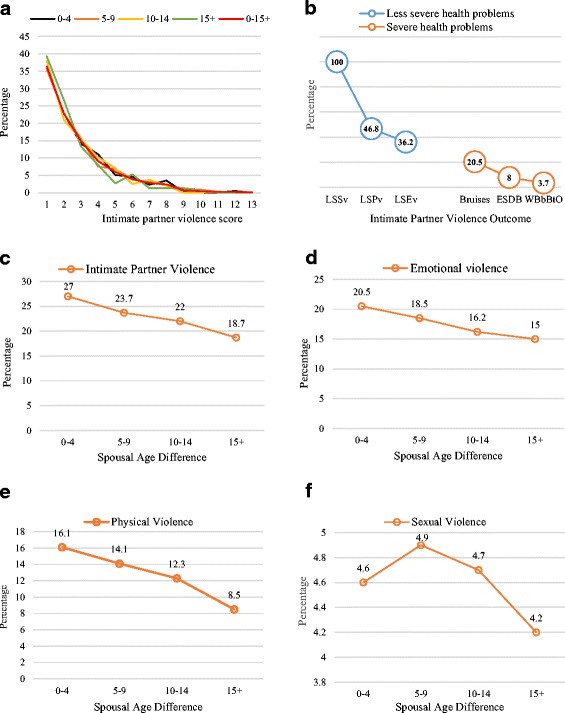
**a** Age difference and IPV among those who experienced IPV. **b** Health problems resulted from domains of violence. **c** Distribution of women by IPV according to spousal age difference. **d** Distribution of women by Emotional violence according to spousal age difference. **e** Distribution of women by Physical violence according to spousal age difference. **f** Distribution of women by Sexual violence according to spousal age difference. *LSSv: Less severe sexual violence; LSPv: Less severe physical violence; LSEv: Less severe emotional violence; ESDB: Eye injuries, sprains, dislocation or bruises; WBbBtO: Wounds, broken bones, broken teeth, or other injuries*

Figure 1a presents the proportion of women who have experienced IPV by spousal age difference. In Fig. 1b, among the women who experienced IPV, all (100%) experienced less severe sexual violence, while 46.8% and 36.2% had experienced less severe physical and emotional violence respectively. For severe health problems as a result of the physical violence, 20.5% had only bruises, 8.0% had one or more of eye injuries, sprains, and dislocations, and 3.7% had either one or more of wounds, broken bones, broken teeth, or other injuries. As shown in Fig. 1c-f, the data show that the proportion of women who had experienced either IPV, emotional, physical or sexual violence reduces with increasing spousal age difference. For any form of violence that had been previously experienced by women, the proportion falls consistently from 27.0% among couples with an age difference of 0–4 years to 18.7% among those whose age difference was at least 15 years. For emotional and physical violence, across all spousal age difference groups, the violence experienced ranged from 25.0%-to-15.0% (*p* < 0.001) and 27.0%-to-18.7% (*p* < 0.001) respectively. In the case of sexual violence, the pattern was different from the patterns exhibited by IPV, physical, and emotional violence. The percentage of women who had experienced sexual violence was 4.6%, 4.9%, 4.7% and 4.2% among couples with the age differences of 0–4, 5–9, 10–14 and 15+ years respectively (*p* > 0.05).

**Fig. 2:**
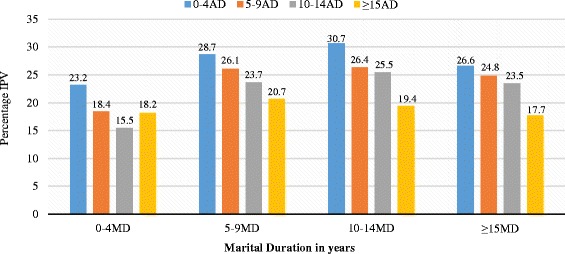
Cross tabulation of Marital Duration (MD) by Spousal Age Difference (AD) according to experienced Intimate Partner Violence. *MD: Marital Duration; AD: Age Difference*

The data as shown in Fig. 2 show that the least IPV was experienced by women who were married 0–4 years prior the survey, irrespective of the spousal age difference. Women whose age difference between them and their partner was 0–4 years experienced the highest IPV across all marital duration categories. Apart from women who have been married for only 0–4 years, the percentage of IPV reduces consistently with increasing spousal age difference.

The data presented in Table 2 show the distribution of women by the type of violence they had experienced from their partners in the past. About 23.5% of the women had experienced any sort of violence, while 18.0%, 13.5% and 4.7% had experienced emotional, physical and sexual violence respectively. The number of children born to such couples was found to be significantly associated with IPV, emotional and physical violence, but insignificant for sexual violence. Across the domains of violence, women who had 3–4 living children experienced more violence than any other childbearing categories. About 28.3% of women who had 3–4 living children had experienced any form of violence, and 22.0% and 16.4% of such women had previously experienced emotional and physical violence respectively. Except for sexual violence, the other domains were not statistically significant; there was an indication that the percentage of women who had experienced violence was higher among women living in urban than in rural areas. While 5.6% of women living in urban areas had experienced sexual violence, 3.1% experienced such violence in the rural areas (*p* < 0.001).

**Table 2:** Percentage Distribution of women by Experienced Violence types

Background	IPV	χ^2^-value	Emotional	χ^2^-value	Physical	χ^2^-value	Sexual	χ^2^-value
Variables		*(p-value)*	Violence	*(p-value)*	Violence	*(p-value)*	Violence	*(p-value)*
Total	23.5 (1589)		18.0 (1218)		13.5 (912)		4.7 (318)	
Number of living children		89.4^a^		64.4^a^		63.2^a^		6.4
None	12.0 (96)	(< 0.001)	9.4 (75)	(< 0.001)	5.9 (47)	(< 0.001)	3.5 (28)	(0.094)
1–2	22.2 (561)		17.1 (431)		12.3 (310)		4.3 (108)	
3–4	28.3 (585)		22.0 (455)		16.4 (338)		5.1 (106)	
5+	25.2 (347)		18.6 (257)		15.7 (217)		5.5 (76)	
Residence		1.89		0.864		3.089		21.8^a^
Urban	24.5 (576)	(0.169)	18.6 (438)	(0.353)	14.5 (341)	(0.079)	3.1 (72)	(< 0.001)
Rural	23.0 (1013)		17.7 (780)		12.9 (571)		5.6 (246)	
Ethnicity		367.5^a^		242.9^a^		304.7^a^		95.5^a^
Hausa/Fulani	11.4 (291)	(< 0.001)	9.4 (241)	(< 0.001)	4.4 (112)	(< 0.001)	2.8 (71)	(< 0.001)
Igbo	28.0 (161)		24.0 (138)		15.3 (88)		4.3 (25)	
Yoruba	24.5 (221)		16.2 (146)		17.2 (155)		1.4 (13)	
Others	33.5 (916)		25.3 (693)		20.4 (557)		7.6 (209)	
Religion		297.5^a^		191.3^a^		359.7^a^		11.3^b^
Christian	33.8 (952)	(< 0.001)	25.4 (715)	(< 0.001)	22.6 (635)	(< 0.001)	5.7 (161)	(0.004)
Muslims	15.8 (605)		12.4 (475)		6.6 (254)		4.0 (153)	
Others	28.8 (32)		25.2 (28)		20.7 (23)		3.6 (4)	
Household wealth		20.1^a^		5.68		20.6^a^		20.5^a^
Poor	20.8 (595)	(< 0.001)	16.8 (480)	(0.058)	11.3 (323)	(< 0.001)	5.6 (161)	(< 0.001)
Middle	25.8 (313)		19.6 (238)		15.4 (187)		5.7 (69)	
Rich	25.3 (681)		18.6 (500)		14.9 (402)		3.3 (88)	
Education		198.4^a^		114.8^a^		246.7^a^		19.5^a^
None	16.0 (467)	(< 0.001)	12.8 (374)	(< 0.001)	7.1 (206)	(< 0.001)	4.0 (116)	(< 0.001)
Primary	32.5 (439)		23.9 (323)		21.9 (296)		6.7 (91)	
Secondary	29.6 (581)		22.5 (441)		18.8 (368)		4.8 (95)	
Higher	19.0 (102)		14.9 (80)		7.8 (42)		3.0 (16)	
Marriage type		0.672		0.616		0.969		0.698
Monogamy	23.7 (1292)	(0.412)	18.1 (990)	(0.432)	13.6 (745)	(0.325)	4.6 (251)	(0.403)
Polygamy	22.6 (285)		17.2 (217)		12.6 (159)		5.1 (65)	
Marital Duration		30.7^a^		23.4^a^		24.2^a^		2.8
0–4	19.0 (336)	(< 0.001)	14.7 (260)	(< 0.001)	10.1 (178)	(< 0.001)	4.1 (73)	(0.425)
5–9	25.8 (412)		20.4 (327)		14.7 (235)		4.5 (72)	
10–14	26.2 (394)		19.9 (299)		14.7 (222)		5.2 (79)	
15+	23.7 (447)		17.6 (332)		14.7 (277)		5.0 (94)	
Husband drinking alcohol		403.8^a^		309.7^a^		502.6^a^		33.6
No	18.5 (1027)	(< 0.001)	14.1 (781)	(< 0.001)	9.1 (501)	(< 0.001)	4.0 (221)	(< 0.001)
Yes	45.4 (560)		35.4 (437)		33.2 (409)		7.9 (97)	
Husband’s Education		159.3^a^		89.9^a^		184.7^a^		8.7^c^
None	15.2 (343)	(< 0.001)	12.3 (278)	(< 0.001)	6.5 (146)	(< 0.001)	4.0 (90)	(0.033)
Primary	30.6 (390)		23.1 (295)		19.6 (250)		6.1 (78)	
Secondary	28.9 (623)		21.4 (462)		18.3 (395)		4.7 (102)	
Higher	21.8 (223)		17.3 (177)		11.2 (114)		4.3 (44)	
Number of marital union		3.133		2.369		2.23^a^		7.0^b^
1	23.0 (1102)	(0.077)	17.7 (845)	(0.124)	13.1 (629)	(0.136)	4.3 (207)	0.008
2+	25.1 (471)		19.3 (362)		14.5 (273)		5.9 (110)	
Empowerment		28.6^a^		16.7^a^		20.7^a^		0.611
Low	21.0 (516)	(< 0.001)	16.1 (397)	(< 0.001)	12.1 (299)	(< 0.001)	4.3 (106)	(0.434)
High	28.1 (486)		21.1 (364)		17.1 (296)		3.8 (66)	

*IPV* Intimate partner violence

^a^Significant at 0.1%; ^b^Significant at 1.0%; ^c^Significant at 5.0%

Ethnicity and religion were significantly associated with all the domains of violence. In the main religious groups in Nigeria, the data show that Igbo women experienced more IPV (28.0%), emotional (24.0%) and sexual violence (4.3%) than did women in the Yoruba and Igbo ethnic groups, but Yoruba women (17.2%) experienced the physical violence among the three ethnic groups. A higher proportion of Christian women than Muslim women experienced IPV (33.8% vs 15.8%), emotional (25.4% vs 12.4%), physical (22.6% vs 6.6%) and sexual violence (5.7% vs 4.0%). The data further show that emotional violence was the only violence domain that was not associated with household wealth. A slight disparity existed between the percentage of women who experienced any type of IPV, physical or sexual violence among those who come from middle-income and rich households, but the margin was wider between middle-class and poor households. The percentage of women who experienced IPV forms a U-shape, with women with no formal education and those with higher education having previously experienced lower levels of violence from their intimate partner than did women who had primary and secondary education. For instance, the prevalence of IPV was 16.0%, 32.5%, 29.6% and 19.0% among those with no formal, primary, secondary and higher education respectively. This was also the pattern exhibited based on the husband’s level of education. Surprisingly, IPV was found to be higher among highly empowered women than those in low level of empowerment.

The percentage of women who reported that they had experienced violence from their intimate partner was strikingly higher among spouses of men who drink alcohol. With regard to previous experiences of any type of IPV, the percentage of women was 45.4% among women whose husbands drank alcohol, compared to 18.5% among women whose husbands did not drink alcohol. In the case of emotional violence, it was 35.1% and 14.1% among women whose husbands drank alcohol and whose husbands were non-drinkers respectively. In the case of physical violence, 33.2% of women whose husbands drank alcohol had experienced such violence, compared to 9.1% of women whose husbands did not drink alcohol.

The data as presented in Table 3 show the results of a bivariate logistic regression that examines the relationship between IPV domains and socioeconomic characteristics. For any violence, the odds reduce consistently, as the spousal age difference increases. The likelihood of IPV was 1.60 (C.*I* = 1.30–1.98, *p* < 0.001), 1.35 (C.*I* = 1.10–1.64, *p* < 0.01) and 1.23 (C.*I* = 0.99–1.52, *p* > 0.05) higher in households where the spousal age differences were 0–4, 5–9 and 10–14 respectively, than among those with a spousal age difference of at least 15 years. In particular, being in spousal age difference groups 0–4 (OR = 2.07; C.*I* = 1.56–2.75, *p* < 0.001), 5–9 (OR = 1.77; C.*I* = 1.34–2.32, *p* < 0.001), and 10–14 (OR = 1.51; C.*I* = 1.13–2.02, *p* < 0.01) years seemed to predispose women to physical violence than among couples where the age difference was 15 years and above. Spousal age difference was not found to be significantly related to sexual violence.

**Table 3:** Unadjusted logistic regression model of experienced violence types according to spousal age difference and background characteristics

Background	IPV	Emotional	Physical	Sexual
Variables	uOR (95% C.I)	uOR (95% C.I)	uOR (95% C.I)	uOR (95% C.I)
Age Difference				
0–4	1.60 (1.30–1.98)^a^	1.46 (1.16–1.84)^b^	2.07 (1.56–2.75)^a^	1.08 (0.71–1.63)
5–9	1.35 (1.10–1.64)^b^	1.29 (1.03–1.60)^c^	1.77 (1.34–2.32)^a^	1.17 (0.79–1.72)
10–14	1.23 (0.99–1.52)	1.10 (0.86–1.39)	1.51 (1.13–2.02)^b^	1.11 (0.73–1.68)
15+	1.00	1.00	1.00	1.00
Number of living children				
None	1.00	1.00	1.00	1.00
1–2	2.09 (1.65–2.63)^a^	1.98 (1.52–2.57)^a^	2.23 (1.62–3.07)^a^	1.23 (0.80–1.88)
3–4	2.87 (2.28–3.65)^a^	2.72 (2.09–3.53)^a^	3.12 (2.27–4.29)^a^	1.49 (0.97–2.27)
5+	2.46 (1.92–3.14)^a^	2.21 (1.67–2.90)^a^	2.98 (2.14–4.14)^a^	1.60 (1.02–2.49)^c^
Residence				
Urban	1.00	1.00	1.00	1.00
Rural	0.92 (0.81–1.04)	0.94 (0.82–1.07)	0.88 (0.76–1.02)	1.87 (1.43–2.45)^a^
Ethnicity				
Hausa/Fulani	1.00	1.00	1.00	1.00
Igbo	3.02 (2.42–3.77)^a^	3.03 (2.40–3.82)^a^	3.94 (2.93–5.30)^a^	1.59 (0.99–2.53)
Yoruba	2.52 (2.07–3.07)^a^	1.85 (1.48–2.31)^a^	4.52 (3.50–5.85)^a^	0.51 (0.28–0.93)^c^
Others	3.92 (3.38–4.53)^a^	3.26 (2.78–3.82)^a^	5.58 (4.51–6.89)^a^	2.90 (2.19–3.81)^a^
Religion				
Christian	1.00	1.00	1.00	1.00
Muslims	0.37 (0.32–0.41)^a^	0.41 (0.36–0.47)^a^	0.24 (0.20–0.28)^a^	0.68 (0.54–0.86)^b^
Others	0.79 (0.52–1.20)	0.99 (0.64–1.53)	0.90 (0.56–1.43)	0.62 (0.22–1.69)
Household wealth				
Poor	1.00	1.00	1.00	1.00
Middle	1.33 (1.13–1.55)^a^	1.21 (1.02–1.44)^c^	1.43 (1.18–1.74)^a^	1.01 (0.75–1.35)
Rich	1.29 (1.13–1.46)^a^	1.13 (0.98–1.29)	1.38 (1.17–1.61)^a^	0.57 (0.43–0.74)^a^
Education				
None	1.00	1.00	1.00	1.00
Primary	2.52 (2.16–2.93)^a^	2.13 (1.80–2.52)^a^	3.69 (3.04–4.47)^a^	1.74 (1.31–2.31)^a^
Secondary	2.20 (1.91–2.53)^a^	1.97 (1.69–2.29)^a^	3.04 (2.53–3.64)^a^	1.23 (0.93–1.62)
Higher	1.23(0.96–1.55)	1.19(0.91–1.54)	1.11(0.79–1.57)	0.74(0.43–1.26)
Marriage type				
Monogamy	1.00	1.00	1.00	1.00
Polygamy	0.94 (0.81–1.09)	0.94 (0.79–1.102)	0.91 (0.75–1.10)	1.13 (0.85–1.49)
Marital Duration				
0–4	1.00	1.00	1.00	1.00
5–9	1.48 (1.25–1.74)^a^	1.49 (1.24–1.79)^a^	1.54 (1.25–1.90)^a^	1.10 (0.78–1.53)
10–14	1.51 (1.28–1.79)^a^	1.44 (1.19–1.73)^a^	1.55 (1.25–1.91)^a^	1.29 (0.93–1.78)
15+	1.33 (1.13–1.56)^b^	1.24 (1.03–1.48)^c^	1.54 (1.25–1.88)^a^	1.22 (0.89–1.67)
Husband drinking alcohol				
No	1.00	1.00	1.00	1.00
Yes	3.64 (3.19–4.15)^a^	3.34 (2.90–3.83)^a^	4.98 (4.28–5.78)^a^	2.05 (1.60–2.62)^a^
Husband’s Education				
None	1.00	1.00	1.00	1.00
Primary	2.47 (2.08–2.91)^a^	2.15 (1.79–2.57)^a^	3.54 (2.84–4.39)^a^	1.57 (1.15–2.15)^b^
Secondary	2.27 (1.95–2.63)^a^	1.94 (1.65–2.29)^a^	3.25 (2.65–3.97)^a^	1.20 (0.89–1.60)
Higher	1.56 (1.29–1.88)^a^	1.50 (1.22–1.84)^a^	1.82 (1.40–2.35)^a^	1.09 (0.75–1.57)
Number of marital union				
1	1.00	1.00	1.00	1.00
2+	1.59 (1.31–1.94)^a^	1.70 (1.37–2.09)^a^	1.73 (1.38–2.17)^a^	1.74 (1.22–2.46)^b^
Empowerment				
Low	1.00	1.00	1.00	1.00
High	1.48 (1.27–1.70)^a^	1.39 (1.18–1.63)^a^	1.50 (1.25–1.78)^a^	0.88 (0.64–1.21)

*IPV* Intimate partner violence*, uOR* Unadjusted odds ratio

^a^Significant at 0.1%; ^b^Significant at 1.0%; ^c^Significant at 5.0%

The risk of IPV was higher among couples who have at least one child than among those without any children. In particular, the odds ratio of IPV was 2.09 (C.*I* = 1.65–2.63, *p* < 0.001), 2.87 (C.*I* = 2.28–3.65, *p* < 0.001) and 2.46 (C.*I* = 1.92–3.14, *p* < 0.001) times more likely among couples who had given birth to 1–2, 3–4 and ≥5 children respectively than among couples who have no children; this pattern was similar to the emotional and physical violence experienced. Among all the types of violence, only the sexual domain shows a significant relationship with place of residence. In this case, the likelihood of sexual violence was found to be higher in the rural areas (OR = 1.87, C.*I* = 1.43–2.45, *p* < 0.001) than in the urban areas. In terms of the relationship between ethnicity and IPV, the odds of IPV, emotional violence and physical violence were lowest among Hausa/Fulani women and highest among Igbo women. However, Yoruba women experienced lower sexual violence (OR = 0.51, C.*I* = 0.28–0.93, *p* < 0.01) than Hausa/Fulani women. In addition, women who practiced the Islamic religion were less likely to experience IPV or any of IPV domains than their Christianity counterparts were. The data further show that the risk of any violence type was significantly higher in households where the husbands drank alcohol. Moreover, the likelihood of IPV, emotional violence and physical violence was 1.48 (C.*I* = 1.27–1.70, *p* < 0.001), 1.39 (C.*I* = 1.18–1.63, *p* < 0.001) and 1.50 (C.*I* = 1.25–1.78, p < 0.001) higher among women with a high empowerment status than among women with a low empowerment status, although there was no significant relationship between empowerment status and sexual violence.

The adjusted models for IPV, EV, PV and SV are shown in Table 4. The data show that spousal age difference was not a significant predictor of IPV and other domains of violence. The identified predictors of IPV were: number of living children, ethnicity, household wealth, woman’s level of education, number of marital unions and husband drinking alcohol. The predictors of emotional violence were: number of living children, ethnicity, woman’s level of education, household wealth, number of marital unions, marital duration and husband drinking alcohol. The predictors of physical violence were: number of living children, ethnicity, household wealth, woman’s level of education, number of marital unions and husband drinking alcohol. And lastly, the predictors of sexual violence were: religion, ethnicity, household wealth, level of education, number of marital unions and husband drinking alcohol.

**Table 4:** Adjusted logistic regression model of experienced violence types according to spousal age difference and background characteristics

Background	IPV	Emotional	Physical	Sexual
Variables	aOR (95% C.I)	aOR (95% C.I)	aOR (95% C.I)	aOR (95% C.I)
Age Difference				
0–4	1.23 (0.89–1.68)	1.15 (0.82–1.61)	1.06 (0.72–1.56)	0.84 (0.54–1.30)
5–9	1.19 (0.88–1.60)	1.20 (0.87–1.65)	1.09 (0.75–1.58)	0.97 (0.65–1.45)
10–14	1.18 (0.86–1.62)	1.06 (0.75–1.49)	1.06 (0.71–1.58)	0.94 (0.61–1.44)
15+	1.00	1.00	1.00	1.00
Number of living children				
None	1.00	1.00	1.00	1.00
1–2	1.78 (1.23–2.56)^b^	1.68 (1.12–2.51)^c^	1.86 (1.14–3.02)^c^	1.16 (0.75–1.78)
3–4	2.37 (1.60–3.51)^a^	2.28 (1.49–3.49)^a^	2.10 (1.25–3.52)^b^	1.39 (0.89–2.14)
5+	2.15 (1.41–3.26)^a^	2.09 (1.32–3.29)^b^	2.16 (1.25–3.71)^b^	1.36 (0.86–2.14)
Residence				
Urban				1.00
Rural				1.25 (0.89–1.75)
Ethnicity				
Hausa/Fulani	1.00	1.00	1.00	1.00
Igbo	1.80 (1.24–2.61)^b^	2.07 (1.39–3.07)^a^	1.49 (0.91–2.43)	2.22 (1.24–3.96)^b^
Yoruba	1.93 (1.40–2.65)^a^	1.52 (1.07–2.16)^c^	2.78 (1.83–4.23)^a^	0.75 (0.39–1.46)
Others	2.83 (2.16–3.71)^a^	2.48 (1.85–3.33)^a^	2.62 (1.80–3.81)^a^	3.03 (2.15–4.26)^a^
Religion				
Christian	1.00	1.00	1.00	1.00
Muslims	1.06 (0.84–1.33)	1.27 (0.98–1.63)	0.78 (0.58–1.03)	1.58 (1.09–2.28)^c^
Others	1.13 (0.61–2.06)	1.36 (0.72–2.55)	1.02 (0.50–2.06)	0.61 (0.21–1.73)
Household wealth				
Poor	1.00	1.00	1.00	1.00
Middle	0.60 (0.46–0.77)^a^	0.56 (0.42–0.73)^a^	0.63 (0.46–0.85)^b^	0.65 (0.46–0.90)^c^
Rich	0.64 (0.49–0.82)^a^	0.58 (0.44–0.76)^a^	0.61 (0.45–0.81)^b^	0.42 (0.28–0.62)^a^
Education				
None	1.00	1.00	1.00	1.00
Primary	1.57 (1.20–2.05)^b^	1.50 (1.12–2.00)^b^	1.91 (1.36–2.67)^a^	1.60 (1.11–2.30)^c^
Secondary	1.55 (1.14–2.08)^b^	1.59 (1.14–2.19)^a^	1.57 (1.08–2.28)^c^	1.70 (1.11–2.60)^c^
Higher	1.01 (0.66–1.52)	1.04 (0.65–1.63)	0.75 (0.43–1.30)	1.44 (0.71–2.89)
Marital Duration				
0–4	1.00	1.00	1.00	
5–9	1.29 (0.99–1.66)	1.35 (1.02–1.78)^c^	1.25 (0.90–1.72)	
10–14	1.13 (0.85–1.49)	1.10 (0.81–1.49)	1.24 (0.87–1.75)	
15+	0.97 (0.72–1.30)	0.91 (0.66–1.24)	1.15 (0.80–1.63)	
Husband drinking alcohol				
No	1.00	1.00	1.00	1.00
Yes	2.25 (1.86–2.73)^b^	2.17 (1.76–2.66)^a^	2.91 (2.33–3.62)^a^	1.77 (1.29–2.43)^a^
Husband’s Education				
None	1.00	1.00	1.00	1.00
Primary	1.13 (0.84–1.49)	1.15 (0.84–1.56)	1.21 (0.83–1.74)	1.20 (0.82–1.75)
Secondary	1.09 (0.81–1.45)	1.10 (0.80–1.50)	1.18 (0.80–1.72)	1.13 (0.75–1.69)
Higher	1.07 (0.76–1.51)	1.27 (0.87–1.83)	1.05 (0.67–1.63)	1.43 (0.87–2.35)
Number of marital union				
1	1.00	1.00	1.00	1.00
2+	1.69 (1.28–2.23)^a^	1.86 (1.40–2.48)^a^	1.91 (1.39–2.61)^a^	1.58 (1.09–2.30)^c^
Empowerment				
Low	1.00	1.00	1.00	
High	1.10 (0.93–1.31)	1.13 (0.94–1.35)	0.99 (0.80–1.21)	
*-2loglikelihood*	*4108.677*	*3635.694*	*2938.242*	*2365.494*
*R Square*	*0.144*	*0.113*	*0.179*	*0.074*
*H-L Test (χ* ^*2*^ *(sig.))*	*3.023 (0.933)*	*6.704 (0.569)*	*8.294 (0.405)*	*3.108 (.927)*

*aOR* Adjusted odds ratio*, H-L* Hosmer and Lemeshow

^a^Significant at 0.1%; ^b^Significant at 1.0%; ^c^Significant at 5.0%

The likelihood of IPV was 1.78c (C.*I* = 1.23–2.56, *p* < 0.01), 2.37 (C.*I* = 1.60–3.51, *p* < 0.001) and 2.15 (C.*I* = 1.41–3.26, *p* < 0.001) higher among couples who had 1–2, 3–4 and 5+ living children respectively, than among those who had never had any children. This statistically significant pattern was also exhibited by physical, emotional violence and number of living children. The Igbo and Yoruba women were more likely to have experienced IPV, emotional and physical violence than were Hausa/Fulani women. The odds of experiencing IPV was 1.80 (C.*I* = 1.24–2.61, *p* < 0.01) and 1.93 (C.*I* = 1.40–2.65, *p* < 0.001) higher among Igbo and Yoruba women respectively than among their Hausa/Fulani counterparts. With regard to previous experiences of physical violence, Yoruba women had a higher risk (OR = 2.78; C.*I* = 1.83–4.23, *p* < 0.001) than did Hausa/Fulani women. Also the chance of sexual violence was higher among Igbo women (OR = 2.22; C.I = 1.24–3.94, *p* < 0.01) than among Hausa/Fulani women. Having been married more than once (OR = 1.58; C.*I* = 1.09–2.30, *p* < 0.05) furthermore predisposes Nigerian women to a higher risk of sexual violence than was the case among those who had only been married once.

The likelihood of IPV was 2.25 (C.*I* = 1.86–2.73, *p* < 0.001) times higher among women whose husbands drank alcohol; this pattern was found across the three domains of violence. The likelihood of IPV, physical violence and sexual violence falls consistently with increasing level of household wealth. While the data showed no significant difference between women with no formal education and those who had a higher level of education with regard to the violence they had experienced, whether IPV, sexual or emotional violence, the risks were significantly higher among women with a primary and secondary level of education than among those with no formal education. The likelihood of IPV was 1.57 (C.*I* = 1.20–2.05, *p* < 0.01) and 1.55 (C.*I* = 1.14–2.08, *p* < 0.01) higher among women with primary and secondary education respectively than among those with no formal education. For sexual violence, the chance was 1.60 (C.*I* = 1.11–2.30, *p* < 0.05) and 1.70 (C.I = 1.11–2.60, *p* < 0.05) higher among women with primary and secondary education than among those with no formal education.

## Discussion

Intimate partner violence is a problem that potentially affects every family, although its severity level varies across socio-cultural characteristics [2, 12]. In Nigeria, as in other nations across the world, IPV most especially against women is still prevalent. Researchers in the areas of gender-based violence have attributed this mainly to the long-standing traditions that give men power and dominance at the household level; they agree that most men who perpetrate such violence do so as one of the means of enforcing their dominance and control [24, 26]. In Nigeria, it is illegal for men to beat their wives physically, but in most situations, IPV cases are not often reported because most women perceive such violence to be a family-based issue that can and should be resolved within the familial structure. Intimate partner violence remains an issue of concern for both researchers and government in Nigeria, because there has been an increase in the number of reported cases in the media and more importantly, a high number of marriages dissolve as a result of IPV. The desire to identify the socioeconomic factors that contribute to IPV was the reason for conducting this study. I therefore examined the relationship between spousal age difference and IPV.

The study revealed that the average spousal age difference was 8.20 ± 5.0 years, and the mean difference was lower among households where wives have experienced some form of IPV. This implies that men who commit IPV are much older than their wives. Traditionally, men marry women who are younger than they are; the persistent harsh economic conditions that have lasted for about four decades in Nigeria have led to men only marrying later in life, which further widens the age gap between couples. The expectation is that IPV should reduce where the spousal age difference is wider, because in such instances, the man is expected to be more mature than his wife, and thus should be able to tolerate some inadequacies of his wife in terms of behavioral attitudes. However, it seems that this expectation is not correct, as reviewed in the theoretical framework in the background section of this paper. Although the odds of IPV significantly reduced as spousal age difference increases, spousal age difference was not found to be a predictor of IPV. This finding is consistent with the outcome of the study conducted in America [19].

In this study, about one in every four women had experienced IPV, while emotional violence was found to be the most prevalent of the domains of IPV, followed by physical and sexual violence in that order. This variation pattern in the prevalence of IPV and its domains echoes the findings of studies previously conducted in Nigeria and some parts of Africa [7, 8, 11, 14]. The injuries of women who had experienced sexual violence appeared to be less severe. In the case of physical violence, about half of the victims have less severe injuries; but for women who reported emotional violence, 1 in 3 women had experienced less severe injuries. In addition, 1 in 5 women who experienced physical violence reported that they had bruises, some had one or more of eye injuries, sprains or dislocations, and only very few had either one or more of wounds, broken bones, broken teeth, or other injuries. The injuries mentioned by those who had experienced violence in this study have been previously reported in the literature too, and the pattern exhibited by the injuries was similar to what was found in earlier studies [7, 9, 14].

The number of years that couples had been married appeared to be important to IPV [30]. The shared marital experience can enhance intimacy and foster better relations between spouses. In new marriages, couples are yet to recognize and accept some of the hidden behaviours and attitudes of their partners, and they need time to learn about these on a daily basis. In this study, IPV was lowest among women who had been married 0–4 years and among those who had been married for at least 15 years prior to the survey, regardless of the spousal age difference. Specifically, being married for 0–4 years seemed to protect spouses from IPV and emotional violence, which increased when they had been married for 5–9 years; however, no significant difference was found among those who had been married for at least 10 years. To some extent, these outcomes agree with the findings from Katerndahl et al.’s study, where it was established that the longer the relationship had lasted, the more predictable and periodic were the dynamics of IPV [30]. In this regard, the longer duration of marriage also tended to increase the number of years of exposure to the risk of IPV, although other socioeconomic situations within the family might also play an important role in reversing such risk.

The chance that a woman would experience violence from her intimate partner was found to increase, as the family size increased; it was lowest among couples who did not have children. The prolonged harsh economic conditions in Nigeria, combined with the high levels of unemployment, which has caused the failure of some men to discharge their responsibilities as head of the household could be responsible for this finding. All things being equal, under the current economic condition in Nigeria, it is assumed that families with a lower number of children may find it easier to meet their immediate needs than families with a larger number of children. Where resources are lacking and when facing numerous family needs that are echoed by the wife, the husband may resort to violence [28]. This is evident in the relationship between household wealth and IPV that was found in this study, which revealed that IPV fell consistently as the household wealth increased. The outcomes of a previous study conducted in Nigeria contradict the direction of association found between family size and IPV in this study [13]. While Aduloju et al.’s study was hospital-based and was conducted in a state in Nigeria, the current study was population-based and used a nationally representative sample. However, the relationship between household wealth and IPV found in this study does corroborate the findings in the literature [12]. It is pertinent to note that the marital duration of couples without any children is likely to be shorter than that of their counterparts who have children, thus the number of years of exposure to the risk of IPV is likely to be higher in the latter than in the former. This may also account for the reason why IPV was least experienced by couples who had no children, as found in this study.

The cultural environment is important when discussing issues relating to IPV [24]. In marriage, the traditions and customs of a society determine the degree of male dominance over their female partners. Nigeria has three main ethnic groups, namely Hausa/Fulani, Igbo and Yoruba among its diverse ethnic clusters. The people in each of these ethnic groups still hold on to their marriage traditions and demands, irrespective of their level of education and modernization. In Nigeria, male dominance is a common practice throughout other areas of life, such as employment, finance, commerce, industry etc., and not only in marriage. Wide age differences in partnership are most common among Hausa/Fulani because girls traditionally marry very early in life. It is a usual practice among Igbo men to marry later in life, because they are required to pay huge dowries as part of the cultural requirements for marriage in Igbo land. The Yoruba tradition does not encourage child marriage and to some extent frowns at the payment of dowry, but it does require a man to be able to meet his family’s needs before getting married. This study showed that IPV, emotional, physical and sexual violence were experienced the least often by Hausa/Fulani women, in comparison to their Igbo and Yoruba counterparts. The possible reason for this finding is that the Hausa/Fulani girls might be too young, weak and immature to protest when their husbands demand something from them, whereas the slightly older and thus more mature women of the Igbo and Yoruba are more likely to say no to unreasonable demands and to stand up for themselves.

Education is a way of breaking the prevalence of male dominance in marriage, irrespective of the spousal age difference. It is known that women who are more educated are also more likely to be aware of their fundamental human rights and thus more empowered within the household in terms of their involvement in family issues/decisions than women who have received less education. The tussle for power and the non-compliance or disagreement of women with some of the rules laid down by the husband may trigger violence within the marriage. As one of the important predictors of IPV in this study, the positive relationship between level of education and IPV, where increasing the level of education was found to be non-protective of IPV, has been established in the literature [31] but the finding is at variance with a similar study conducted in Bangladesh [32]. The context-specific differences between the Bangladesh study and the current study could explain this difference. In addition, the husbands’ alcohol consumption was found in this study to be important predictor of violence in all violence domains. Women in families where husbands consumed alcohol experienced higher IPV, emotional, physical and sexual violence than did women those husbands did not drink alcohol. This finding corroborates the outcome of earlier studies on the relationship between husband’s alcohol intake and IPV [33].

### Limitations of the study

This study had some limitations. Firstly, it is likely that IPV cases are under-reported, because women are often afraid or reluctant to report it, particularly among low-income groups. It is also likely that some communities may have reservations about reporting IPV in general. These certainly will have an implication on the prevalence of IPV found in this study. Therefore, our results may not be representative of all cases of IPV and may be biased towards populations with more sensitization towards IPV or a greater awareness of programmes relating to IPV. According to the findings of this study, however, more efforts are needed to reduce the level of IPV, as stipulated by the SDGs. Secondly, it is also likely that the number of IPV cases was grossly under-reported because women who were hospitalized as a result of experiencing violence during the course of the survey, were not captured due to the population-based nature of this study. Thirdly, the cross-sectional nature of this study suggests that causality cannot be clearly established, and therefore the readers of this article should interpret the findings with caution. Lastly, family arrangement has a direct relation to IPV, but that has not been covered because the data did not capture information that could be used for such analysis.

## Conclusion

The level of IPV found in this study was high; it appeared to reduce with increasing spousal age difference. Spousal age difference is not a predictor of IPV, but a higher spousal age difference was found to be protective of IPV. Therefore, this study recommends that men need to be sufficiently mature before entering into marriage, as this will reduce the level of IPV in Nigeria. Strategies to eradicate IPV in Nigeria should target couples with lower age differences as well as families where the husband drinks alcohol. While the age difference may not really be an important factor in examining IPV amidst other covariates, the predictors of IPV found in this study were: family size, ethnicity, household wealth, education, marital duration and husband’s alcohol consumption. These factors should be taken into consideration when designing frameworks on reducing IPV in Nigeria. The findings underscore the risk associated with the influence of spousal age difference on IPV. Context-specific qualitative studies are needed in Nigeria to explore the relationship between partner age differences and IPV further.
